# Characterization of a synthetic bacterial self-destruction device for programmed cell death and for recombinant proteins release

**DOI:** 10.1186/1754-1611-5-8

**Published:** 2011-06-07

**Authors:** Lorenzo Pasotti, Susanna Zucca, Manuel Lupotto, Maria Gabriella Cusella De Angelis, Paolo Magni

**Affiliations:** 1 Dipartimento di Informatica e Sistemistica, Università degli Studi di Pavia, via Ferrata 1, Pavia, Italy; 2 Centro di Ingegneria Tissutale, Università degli Studi di Pavia, via Ferrata 1, Pavia, Italy

## Abstract

**Background:**

Bacterial cell lysis is a widely studied mechanism that can be achieved through the intracellular expression of phage native lytic proteins. This mechanism can be exploited for programmed cell death and for gentle cell disruption to release recombinant proteins when *in vivo *secretion is not feasible. Several genetic parts for cell lysis have been developed and their quantitative characterization is an essential step to enable the engineering of synthetic lytic systems with predictable behavior.

**Results:**

Here, a BioBrick™ lysis device present in the Registry of Standard Biological Parts has been quantitatively characterized. Its activity has been measured in *E. coli *by assembling the device under the control of a well characterized N-3-oxohexanoyl-L-homoserine lactone (HSL) -inducible promoter and the transfer function, lysis dynamics, protein release capability and genotypic and phenotypic stability of the device have been evaluated. Finally, its modularity was tested by assembling the device to a different inducible promoter, which can be triggered by heat induction.

**Conclusions:**

The studied device is suitable for recombinant protein release as 96% of the total amount of the intracellular proteins was successfully released into the medium. Furthermore, it has been shown that the device can be assembled to different input devices to trigger cell lysis in response to a user-defined signal. For this reason, this lysis device can be a useful tool for the rational design and construction of complex synthetic biological systems composed by biological parts with known and well characterized function. Conversely, the onset of mutants makes this device unsuitable for the programmed cell death of a bacterial population.

## Background

Naturally occurring lytic and temperate bacteriophages have the ability to provoke the host cell lysis through the expression of specific proteins during the lytic cycle. In many phages, like the T4 phage and the lambda phage, these proteins have been identified and widely studied [1-4]. In particular, holins form stable and non-specific lesions in the cytoplasmic membrane that allow the lysozymes to gain access to the peptidoglycan layer. Lysozymes are generally soluble proteins with one or more muralytic activities against the three different types of covalent bonds (glycosidic, amide, and peptide) of the peptidoglycan polymer of the cell wall [5,6]. The combined work of holin and lysozyme results in the degradation of the two cell membranes of gram-negative bacteria, thus causing cell lysis. Antiholin is a third protein involved in this process as it inhibits holin and is responsible for the regulation of its activity [7].

The described lytic mechanism can be exploited for the release of useful recombinant proteins which cannot be secreted by the engineered host strain [8].

*Escherichia coli *is a widely used organism for recombinant protein production, but its secretion capabilities are limited and recombinant protein targeting to the growth medium has shown to work only with a small set of proteins [9]. For this reason, cell disruption techniques are required to gain the intracellularly expressed protein of interest. Mechanical techniques, such as cell ultrasonication, usually result in protein denaturation caused by the heat produced during the process and some of them are also unfeasible on industrial scale, whereas non-mechanical techniques, such as chemical membrane degradation with detergents or enzymes, involve the purchase of expensive reagents [8]. Other recently proposed mechanical, chemical, physical and enzymatic treatments to disrupt the cell membrane, especially focused on high throughput screening, are reviewed in [10]. The engineering of a lysis system that is triggered by a user-defined signal can avoid the use of common cell disruption techniques for the recovery of intracellularly expressed proteins.

Another important application of an inducible lytic system is the programmed cell death of a bacterial population, which might be useful in those processes where the microorganism must be eliminated at a specific time, after having completed its work.

In literature, inducible lysis systems have been proposed. T4 phage holin and T7 phage lysozyme genes have been used to construct lytic *E. coli *strains to achieve the gentle disruption of cells upon IPTG induction using the *lac *promoter or the DE3 inducible system [8,11]. The T7 lysozyme was used to both cut bonds in the cell wall and tightly regulate holin gene by inhibiting the T7 polymerase basal expression in uninduced DE3 inducible system. The same genes have been used under the control of a glucose starvation-inducible promoter to allow cell autolysis upon glucose exhaustion in the medium [12]. Heat- and UV-inducible promoters have been used to regulate the lambda phage lysis cassette *SRRz *for high throughput enzyme release [13].

**Figure 1:**
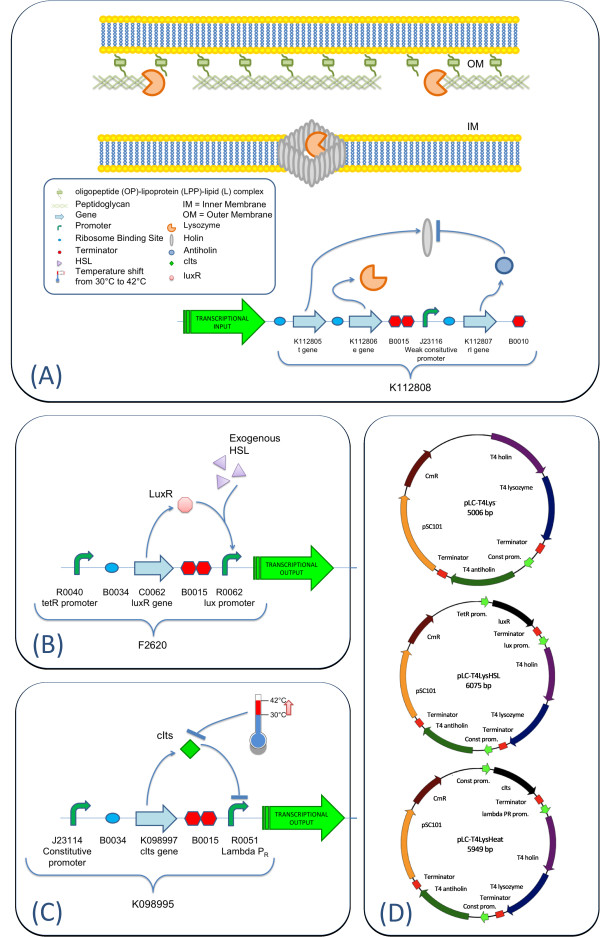
**Overview of the devices used in this work**. The gram-negative bacteria envelope consists in an inner membrane, an outer membrane and a peptidoglycan layer, which is linked to the outer membrane via oligopeptide (OP)-lipoprotein (LPP)-lipid (L) complexes and gives structural strength to the cell wall [25]. The lysis device is composed by a promoterless operon encoding a holin (gene *t*) and a lysozyme (gene *e*). When the operon is expressed, holin forms lesions in the inner membrane. Lysozyme can pass through these lesions, reaching and attacking the peptidoglycan layer, thus achieving cell lysis. In addition, the weak promoter BBa_J23116 constitutively expresses gene *rI*, encoding an antiholin, which inhibits the holin action caused by basal expression of the *t-e *operon (A). BBa_F2620 HSL-inducible input device is composed by a *luxR *expression cassette, driven by the *tetR *promoter, and the *lux *promoter, which is normally off. Its transcription can be induced by the LuxR transcription factor in presence of exogenously added HSL (B). BBa_K098995 heat-inducible input device is composed by a *cIts *expression cassette, driven by the BBa_J23114 constitutive promoter, and PR repressible promoter from lambda phage. cIts is a heat-sensitive repressor of PR: when temperature is 30°C the repressor tightly keeps the promoter in the off-state, while a temperature shift to 42°C can induce the transcription of PR (C). Plasmid maps of the promoterless lysis device pLC-T4Lys^-^, the HSL-inducible lysis device pLC-T4LysHSL and the heat-inducible lysis device pLC-T4LysHeat, all of them in a low copy number vector (D).

The MIT Registry of Standard Biological Parts [14] hosts several lysis protein coding sequences and devices, but most of them, despite their important potential applications, remain uncharacterized. The BioBrick™ device^1 ^BBa_K112808 consists of a promoterless operon composed by the T4 phage genes *t *and *e*, encoding a holin and a lysozyme respectively [15]. Downstream of the transcriptional terminator of the operon, the T4 *rI *gene, encoding a *t*-specific antiholin, is present under the control of a weak constitutive promoter (see Figure 1A for a detailed overview of this lysis device). The T4 antiholin is able to heterodimerize with the *t *gene product, thus avoiding the holin to form pores in the inner membrane [16]. When the lysis device is assembled under the control of an inducible promoter, a weak constitutive expression of *rI *prevents cell lysis, which may be caused by the basal expression of the promoter in the off-state, and in this way the tight regulation of the inducible lysis can be obtained.

In this work, BioBrick™ parts have been used to quantitatively characterize the BBa_K112808 lysis device by assembling it to BBa_F2620, a well characterized N-3-oxohexanoyl-L-homoserine lactone (HSL)-inducible device based on the *lux *promoter (see Figure 1B for a working diagram of this device) [17], thus yielding the new BioBrick™ BBa_K173015. BBa_F2620, whose transfer function has already been measured, has been used to drive the expression of BBa_K112808 over a range of transcriptional input values and to characterize the lysis behavior as a function of the transcriptional strength in *E. coli*. Finally, in order to study the modularity of this device, a different inducible input device, which can be triggered by heat induction (see Figure 1C for a working diagram of this device), has been assembled upstream, thus yielding the new BioBrick™ BBa_J107014, and the validation of this composite part has been carried out. Figure 1D shows the maps of the three main plasmids used in this work, containing the promoterless lysis device (pLC-T4Lys^-^), the HSL-inducible lysis device (pLC-T4LysHSL) and the heat-inducible lysis device (pLC-T4LysHeat) respectively, all of them in a low copy number vector.

## Methods

### Strains and plasmids

All the *E. coli *strains and plasmids used in this study are listed in Table 1. The BioBrick™ codes reported in the table are given according to the Registry of Standard Biological Parts [14].

**Table 1:** Plasmids and strains used in this study

Strains		
Name	Genotype	Source
TOP10	F- mcrA Δ(mrr-hsdRMS-mcrBC) φ80lacZΔM15 ΔlacX74 nupG recA1 araD139 Δ(ara-leu)7697 galE15 galK16 rpsL(Str^R^) endA1 λ^-^	Invitrogen
DB3.1	F- gyrA462 endA1 glnV44 Δ(sr1-recA) mcrB mrr hsdS20(r_B_^-^, m_B_^-^) ara14 galK2 lacY1 proA2 rpsL20(Sm^r^) xyl5 Δleu mtl1	Invitrogen
DH5alpha	F- endA1 glnV44 thi-1 recA1 relA1 gyrA96 deoR nupG φ80dlacZΔM15 Δ(lacZYA-argF)U169, hsdR17(rK- mK+), λ^-^	Invitrogen
MG1655	F- λ- ilvG- rfb-50 rph-1	CGSC
		
Plasmids		
**Name**	**BioBrick™ code**	**Description**
pLC-ccdB	pSB4C5(BBa_I52002)^a^	pUC19-derived pMB1 replication origin and ccdB toxin constitutive expression cassette in low copy vector
pHC-T4Lys^-^	pSB1A2(BBa_K112808)^a^	promoterless lysis device in high copy vector
pHC-HSL	pSB1A2(BBa_F2620)^b^	HSL-inducible promoter in high copy vector
pHC-RFP	BBa_J61002(BBa_J23118)^a^	RFP constitutive expression cassette
pHC-Heat	pSB1A2(BBa_ K098995)^a^	heat-inducible promoter in high copy vector
pHC-T4LysHSL	pSB1A2(BBa_K173015)^c^	HSL-inducible lysis device in high copy vector
pLC-HSL	pSB4C5(BBa_F2620)^c^	HSL-inducible promoter in low copy vector
pLC-T4Lys^-^	pSB4C5(BBa_K112808)^c^	promoterless lysis device in low copy vector
pLC-T4LysHSL	pSB4C5(BBa_K173015)^c^	HSL-inducible lysis device in low copy vector
pLC-T4LysHeat	pSB4C5(BBa_J107014)^c^	heat-inducible lysis device in low copy vector

^a ^taken from the Registry DNA Distribution 2009

^b ^given by the iGEM Headquarters, Massachusetts Institute of Technology, Cambridge, USA

^c ^constructed in this study

The BioBrick™ code of the plasmids include the vector name (out of brackets) and insert name (in brackets). The prefix pHC- indicates a high copy vector backbone (pSB1A2 or BBa_J61002), while pLC- indicates a low copy vector backbone (pSB4C5).

pSB1A2 and BBa_J61002 are high copy number vectors with a pUC19-derived pMB1 replication origin (~100-300 molecules per cell) and Ampicillin resistance marker, while pSB4C5 is a low copy number vector with a pSC101 replication origin (~5 molecules per cell) and Chloramphenicol resistance marker [18]. The full description of vectors and inserts, including their nucleotide sequence, can be found in the BioBrick™ individual pages in the Registry of Standard Biological Parts web site.

### Cloning methods

Chemically competent TOP10 *E. coli *(Invitrogen) were routinely used both for cloning and for quantitative experiments. Chemically competent DB3.1 *E. coli *(Invitrogen) were used to propagate pLC-ccdB plasmid, containing a ccdB expression cassette, which is toxic for TOP10 but not for DB3.1. DH5alpha (Invitrogen) and MG1655 (purchased from CGSC, Yale University, USA) were only used for quantitative experiments. TOP10, DH5alpha and DB3.1 were heat shock-transformed according to the manufacturer's protocol. MG1655 were made chemically competent with the protocol described in [19] and they were heat-shock transformed at 42°C with the required plasmid. All the strains were routinely grown at 37°C in selective LB medium [19] with Ampicillin (100 μg/ml) or Chloramphenicol (12.5 μg/ml) to propagate plasmids, except pLC-T4LysHeat that was grown at 30°C to avoid heat-induction of lysis genes. Plasmids have been purified through QIAprep Spin Miniprep kit (Qiagen) from 5 ml overnight cultures. For every plasmid, 750 μl of culture were mixed with 250 μl of sterile 80% glycerol solution for long-term storage at -80°C. DNA was digested with EcoRI, XbaI, SpeI or PstI according to BioBrick™ Standard Assembly procedure [20] and isolated from 1% agarose gel by Gel Extraction Kit (Roche Diagnostics). Cloning of parts was assessed by T4 Ligase and ligation products were heated at 65°C for 10 min to inactivate T4 Ligase before proceeding with heat shock transformation. All the enzymes were purchased from Roche Diagnostics and used according to manufacturer's protocol.

TOP10-rfp-lys strain (see Protein release assays section), which bears two plasmids, pHC-RFP and pLC-T4LysHSL, was built up with the following procedure: competent TOP10 were transformed with pLC-T4LysHSL, then transformed cells were grown in selective LB and made chemically competent again with the protocol described in [19] and heat shock-transformed at 42°C with pHC-RFP. Co-transformants were selected using LB medium with Ampicillin at 100 ug/ml and Chloramphenicol at 12.5 ug/ml.

LB and M9 supplemented medium (11.28 g/L M9 salts, 1 mM thiamine hydrochloride, 2 mM MgSO4, 0.1 mM CaCl2, 0.2% casamino acids and 0.4% vol/vol glycerol as carbon source) [19] were used in quantitative experiments.

DNA sequencing was performed through BMR Genomics (Padova, Italy) DNA analysis service.

### Plasmid construction

pSB4C5 low copy vector bulk, obtained from pLC-ccdB after EcoRI-PstI cut, was ligated to the inserts of pHC-T4Lys^- ^and pHC-HSL cut with EcoRI-PstI to yield pLC-T4Lys^- ^and pLC-HSL respectively. pHC-T4LysHSL was constructed by assembling the insert of pHC-HSL cut with EcoRI-SpeI to pHC-T4Lys^- ^cut with EcoRI-XbaI. pLC-T4LysHSL was constructed by assembling the insert of pHC-T4Lys^- ^cut with XbaI-PstI to pLC-HSL cut with SpeI-PstI. Finally, pLC-T4LysHeat was constructed by assembling the insert of pHC-Heat cut with EcoRI-SpeI to pLC-T4Lys^- ^cut with EcoRI-XbaI.

### Lysis assays

Unless otherwise noted, 5 μl of bacteria bearing pLC-T4LysHSL and pLC-T4Lys^- ^glycerol stocks were inoculated in 5 ml of selective LB medium and grown at 37°C, 220 rpm overnight. The cultures were diluted 1:100 in 5 ml of selective LB medium and grown for additional 4-5 hours under the same conditions as before. After that time, a 200 μl aliquot of each culture was transferred in a flat-bottomed 96-well microplate (Greiner) and the OD600 was measured with an Infinite F200 microplate reader (Tecan). Based on this measurement, the cultures were diluted to the same OD600 (0.05-0.13) and then six 200 μl aliquots of each culture were transferred in a flat-bottomed 96-well microplate (Greiner). Unless otherwise noted, three wells of each culture were induced with 2 μl of properly diluted HSL (Sigma Aldrich #K3007) and 2 μl of deionized water were added to the other three wells (uninduced wells). If the HSL-inducible lysis device had to be assayed with different HSL concentrations in the same experiment, three 200 μl aliquots of the cultures for each investigated concentration were transferred in the microplate and induced. The microplate was incubated at 37°C in the Infinite F200 microplate reader and assayed every 5 min following this protocol immediately before the measurement: 15 s of linear shaking (amplitude = 3 mm), wait for 5 s, OD600 measurement.

For lysis assays on pLC-T4LysHeat, the cultures were grown at 30°C instead of 37°C and an automatic temperature shift from 30°C to 42°C was used to induce lysis in the microplate reader instead of HSL.

### Analysis of growth curves

Raw OD600 values measured in the Infinite F200 microplate reader were normalized by subtracting for each time point the mean raw absorbance of the media to compute the actual bacterial optical density.

The growth phases of bacterial cultures with the different plasmids used in this study, grown in LB medium in a microplate, have been characterized in each experiment by computing the natural logarithm of the OD600 values *ln(OD600(t)) *over time. Then, the exponential phase was identified by visual inspection as the linear region of *ln(OD600(t))*, the late stationary phase as the constant region and the early stationary phase as the region between the other two.

In all the lysis assays, uninduced bacteria doubling time in exponential growth phase was evaluated by performing linear regression over the *ln(OD600(t)) *linear region to estimate the curve slope *m*, which represents the growth rate of the culture. Then the culture doubling time was computed as *ln(2)/m*.

The lysis entity after induction was computed as 100*(1-min_OD600_/ref_OD600_), where *min_OD600 _*is the minimum OD600 reached by the culture and *ref_OD600 _*is the OD600 immediately before the density drop caused by cell lysis. The rise time was computed as the time required for the 100*(1-OD600(t)/refOD_600_) signal to rise from 10% to 90% of the lysis entity.

### Protein release assays

5 μl of TOP10-rfp-lys, TOP10 bearing pHC-RFP and TOP10 bearing pLC-T4Lys^- ^glycerol stocks were inoculated in 5 ml of selective LB medium and grown at 37°C, 220 rpm overnight. TOP10-rfp-lys and TOP10 with pHC-RFP cultures were diluted 1:100 into six 15 ml tubes containing 5 ml of pre-warmed selective LB medium. TOP10 with pLC-T4Lys^- ^culture was diluted 1:100 in one 15 ml tube with 5 ml of pre-warmed selective LB. The 13 resulting cultures were grown to an OD600 of about 0.55 (exponential phase). Three of the six replicates of TOP10-rfp-lys and TOP10 with pHC-RFP were induced with HSL at a final concentration of 100 nM and all the 13 tubes were incubated under the same conditions as before for 125 min. Every 25 min, the OD600 was measured with the NanoDrop ND-1000 and a 300 μl aliquot was taken from each culture. The 300 μl samples were centrifuged at 11000 rpm in a table top centrifuge and the fluorescence of 200 μl of the supernatant was measured in a microplate with the Infinite F200 reader using: 535 nm excitation filter, 620 nm emission filter, excitation bandwidth = 25 nm, emission bandwidth = 20 nm, gain = 55, number of flashes = 25, integration time = 20 μs, top reading. The raw fluorescence measurements of TOP10-rfp-lys and TOP10 bearing pHC-RFP (induced and uninduced) were normalized by subtracting the background fluorescence value of pLC-T4Lys^- ^cells at the same time points.

To measure the percentage of the RFP released in the medium after 125 minutes, TOP10-rfp-lys, TOP10 bearing pHC-RFP and TOP10 bearing pLC-T4Lys^- ^were grown and induced as described above, in 3 ml cultures. At t = 125 min, the cultures were centrifuged (4000 rpm, 4°C for 10 min) and supernatants and pellets were processed to measure the amount of intracellular and extracellular RFP. Pellets were resuspended in 200 μl of sterile LB broth and 200 μl of 2X lysis buffer (25 mM of Tris-HCl at pH 8.0, 8% SDS) were added to the resuspended pellets [21]. The resuspended pellets with the lysis buffer were left at room temperature for 15 min, then transferred in a 1.5 ml tube and centrifuged at 13000 rpm for 15 min in a table top centrifuge to spin down the cell debris. 200 μl of these supernatants were transferred in a microplate and the RFP fluorescence was measured as described above, to evaluate the intracellular RFP amount. To evaluate the extracellular RFP concentration, the growth media were treated as follows: 100 μl of media were mixed with 100 μl of 2X lysis buffer, left at room temperature for 15 min, transferred in a microplate and the fluorescence measured in the same way as the lysed pellets. The 2X lysis buffer was added to reproduce the same conditions as the pellets. The raw intracellular and extracellular RFP measurements were normalized by subtracting the background fluorescence of pLC-T4Lys^- ^lysed pellet and supernatant respectively. Finally, the mean RFP values of lysed pellets and supernatants of TOP10-rfp-lys and TOP10 bearing pHC-RFP (induced and uninduced) were corrected by the total culture volume, so the percentage of extracellular RFP molecules was computed for each culture (see Additional file 1, Supplementary Methods and Supplementary Table 1 for details).

### Optical density calibration

OD600 measurements performed with the Infinite F200 microplate reader and the NanoDrop ND-1000 were converted to OD600 measurements in 1 cm pathlength by calibrating the two instruments with the V-530 spectrophotometer (Jasco), measuring the OD600 of serial dilutions of a TOP10 culture grown in LB or M9 supplemented medium.

### Evolutionary stability characterization

Bacteria bearing pLC-T4LysHSL were propagated for 100 generations as described in [22] without adding HSL and every 10 generations a lysis assay was performed to test the stability of the lysis phenotype. Stability was assessed by measuring lysis entity in each assay. In particular, in order to achieve 100 generations, the culture was diluted 1:1000 every 24 hours, thus yielding about 10 generations per day (log_2_1000 = 9.97) [22]. Every day, an aliquot was taken from the propagated culture and lysis was assayed as described above. This experiment was performed in triplicate for each tested strain.

### Analysis of mutants

To analyze mutants, a lysis assay was performed on TOP10 bearing pLC-T4LysHSL induced with HSL 10 nM and when the lysed and re-grown cells reached an OD600 = 0.22 they were diluted 1:1000 in fresh selective LB medium in a new 96-well microplate, incubated in the Infinite F200 reader and induced again with HSL 10 nM when they reached an OD600 of 0.35 (exponential phase) to check if they could lyse again. The re-grown cells were also streaked on selective LB agar, then 2 single colonies were inoculated in 5 ml of selective LB and let grow overnight (37°C, 220 rpm). Plasmid DNA was extracted from the 5 ml overnight cultures and analyzed by restriction enzyme digestion/electrophoresis and DNA sequencing using primers VF2: 5'-TGCCACCTGACGTCTAAGAA-3', VR: 5'-ATTACCGCCTTTGAGTGAGC-3' and C0062VF: 5'-GAATGTTTAGCGTGGGCATG-3'. This procedure was carried out starting from 5 μl of pLC-T4LysHSL glycerol stock and also from single colonies isolated from this stock.

## Results and Discussion

### Lysis assays

Lysis was assayed in a 96-well microplate by measuring the optical density at 600 nm (OD600) dynamics of TOP10 bearing HSL-inducible lysis device pLC-T4LysHSL, induced with HSL and uninduced. Induced and uninduced TOP10 bearing the promoterless lysis device pLC-T4Lys^- ^were used as a negative control in all the experiments. Lysis entity, i.e. the maximum percent reduction of OD600 caused by lysis induction, was computed in all the assays as an indirect measurement of the amount of lysed cells.

**Figure 2:**
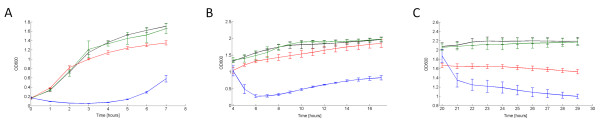
**Lysis profile of TOP10 bearing the lysis device in low copy number when induced in different growth phases in microplate reader**. OD600 of TOP10 with pLC-T4LysHSL induced with HSL 100 nM (blue line) and uninduced (red line), pLC-T4Lys^- ^induced with HSL 100 nM (green line) and uninduced (black line). Induction was performed in exponential phase (OD600 = 0.2) at t = 0 (A), early stationary phase (OD600~1.3) at t = 4 h (B) and late stationary phase (OD600~2) at t = 20 h (C). Error bars represent the 95% confidence interval of the estimated mean. For clarity of presentation, data points shown here are resampled with a 1-hour sampling time.

A typical lysis profile is reported in Figure 2, where TOP10 bearing pLC-T4LysHSL were induced with HSL 100 nM at t = 0 h (OD600 = 0.2, exponential phase), t = 4 h (OD600~1.3, early stationary phase) or t = 20 h (OD600~2, late stationary phase). Lysis began after about 15 min from the induction in all the growth phases and its mean entity was about 76.3 ± 0.3%, 75.4 ± 1.1% and 50 ± 2.5% at t = 0 h, t = 4 h and t = 20 h, respectively (Table 2).

**Table 2:** Quantitative characterization of TOP10 bearing the HSL-inducible lysis device in low copy plasmid grown at 37°C in microplate

	TOP10 with pLC-T4LysHSL		
	Exponential phase	Early stationary phase	Late stationary phase
Lysis entity [%]	76.28 ± 0.3	75.43 ± 1.1	50.1 ± 2.5
Lysis delay after induction [min]	15	15	15
Doubling time [min]	49.8 ± 1.1		
Doubling time of negative control [min]	43 ± 1.3		

Induction was carried out with HSL 100 nM. Mean lysis entity and lysis delay after the induction are reported for the different growth phases together with their standard error, measured on 3 independent experiments. The doubling time of the uninduced lysis device and its negative control are reported too.

**Figure 3:**
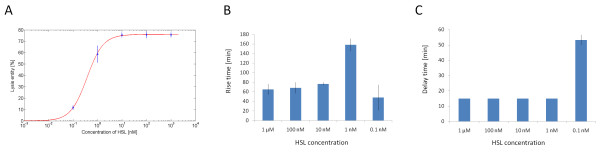
**Transfer function, rise time and delay time of the HSL-inducible lysis device in low copy plasmid in early stationary phase in microplate reader**. Lysis entity of TOP10 cells with pLC-T4LysHSL induced with different concentrations of HSL in early stationary phase at OD600 = 0.9 (A). The experimental data (circles) were fitted with a Hill function (line, Vmax = 76, K_50 _= 0.37, n = 1.3). For each concentration, the rise time, i.e. the time to rise from the 10% to 90% of the lysis entity (B) and the delay time before the OD600 drop after induction (C) are also shown. Error bars represent the 95% confidence interval of the estimated mean.

The mean doubling time of uninduced TOP10 bearing pLC-T4LysHSL, evaluated on all the experiments, was 49.8 ± 1.1 min, while the doubling time of the uninduced negative control pLC-T4Lys^- ^was 43 ± 1.3 min, thus demonstrating that the HSL-inducible lysis device gives a reasonably low metabolic burden and allows the cells to grow at a rate comparable to their negative control (Table 2). In all cases, after about 2-3 h from the induction, cells start growing again, suggesting the onset of mutants that have lost the inducible lysis phenotype. Figure 3A shows the measured transfer function of pLC-T4LysHSL induced at t = 4 h (OD = 0.9, early stationary phase) with different HSL concentrations. The rise time, i.e. the time required to rise from 10% to 90% of the lysis entity, and the delay time before the OD600 drop are also reported for each HSL concentration (Figure 3B and 3C). As these parameters show, the lysis dynamics is highly nonlinear, in fact the delay and rise times change as a function of the induction entity. In particular, the delay time is equal for all the HSL concentrations except for the smallest one (0.1 nM), in which it is about 40 min longer than measured for the other concentrations. The rise time increases as a function of the HSL concentration, reaching its highest value at 1 nM HSL (more than 2.5 hours). However, at 0.1 nM the rise time is less than 1 hour.

Analogous studies were conducted for the HSL-inducible lysis device in high copy plasmid. However, for its evident instability and for the too high doubling time of uninduced cells (see Additional file 1, Supplementary Results for details), the lysis device in high copy plasmid has not been considered for further studies.

**Figure 4:**
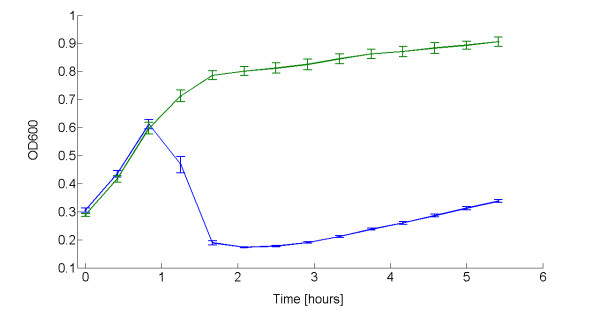
**Lysis dynamics of TOP10 bearing the thermoinducible lysis device in low copy plasmid grown in microplate reader**. OD600 of TOP10 with pLC-T4LysHeat induced with a temperature shift from 30°C to 42°C in the microplate reader (blue line). Heat-induced pLC-T4Lys^- ^(green line) is shown as the negative control. Induction was performed in exponential phase at OD600 = 0.3. Error bars represent the 95% confidence interval of the estimated mean. For clarity of presentation, data points shown here are resampled with a 30-minute sampling time.

Lysis was also assayed for TOP10 bearing pLC-T4LysHeat low copy vector containing a heat-inducible lysis device. Induction was triggered in a 96-well microplate by shifting the incubation temperature from 30°C to 42°C. The results are shown in Figure 4 and summarized in Table 3. The lysis entity was comparable to the one in the HSL-inducible device in low copy. The doubling time of the heat-inducible lysis device was similar to the negative control, but both were much higher than those reported in Table 2 because cultures were grown at 30°C instead of 37°C, causing a slower growth rate. Also the time delay after induction in the thermoinducible lysis device is much higher than in the HSL-inducible device. This should be due to the different response time of the two input devices.

**Table 3:** Quantitative characterization of TOP10 bearing the thermoinducible lysis device in low copy plasmid grown at 30°C in microplate

TOP10 with pLC-T4LysHeat	
Lysis entity in exponential phase [%]	73 ± 0.3
Lysis delay after induction [min]	55
Doubling time [min]	66.1 ± 1.7
Doubling time of negative control [min]	65.9 ± 0.6

Induction was carried out by shifting the temperature from 30°C to 42°C. Mean values estimated on three wells in the same experiment are reported with standard errors.

Experiments on pLC-T4LysHSL and pLC-T4LysHeat were also performed in a different growth medium (M9 with glycerol as carbon source, supplemented with thiamine and casamino acids) and in two other *E. coli *strains (DH5alpha and MG1655) grown in LB, giving consistent results when compared to the TOP10 strain in LB medium results described in this section (see Additional File 1, Supplementary Figure 1, Supplementary Figure 2 and Supplementary Table 3 for a detailed description of the results).

### Protein release assays

A co-transformed TOP10 strain (here called TOP10-rfp-lys) bearing both a high copy plasmid with a Red Fluorescent Protein (RFP) constitutive expression cassette pHC-RFP and the HSL-inducible lysis device pLC-T4LysHSL was induced with HSL to study the RFP release in the medium. Uninduced TOP10-rfp-lys, TOP10 bearing pHC-RFP (induced and uninduced) and TOP10 bearing pLC-T4Lys^- ^were chosen as controls in this assay.

**Figure 5:**
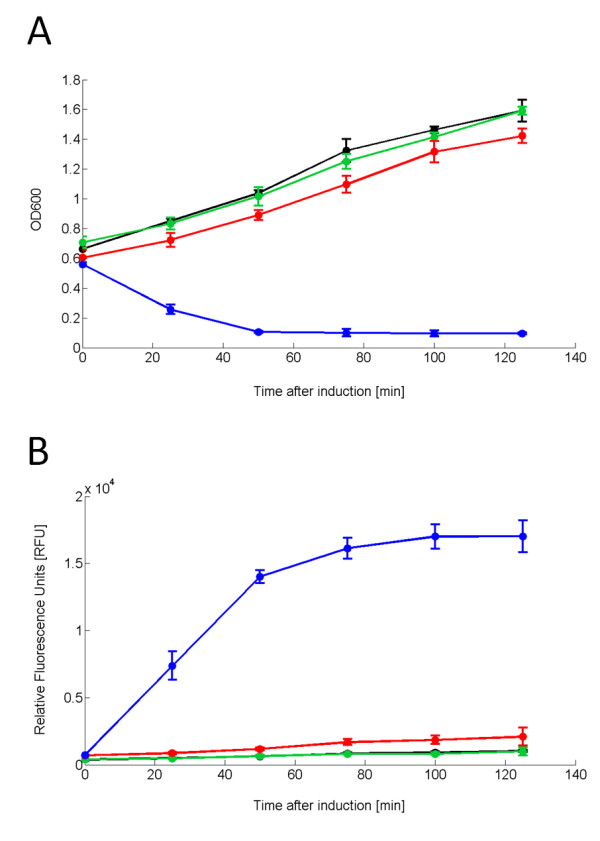
**OD600 time course of TOP10-rfp-lys grown in 15 ml tubes upon induction with HSL 100 nM (A) and RFP fluorescence time course in the supernatant (B)**. Culture absorbance (A) and supernatant fluorescence (B) of TOP10-rfp-lys induced with HSL 100 nM (blue line). Uninduced TOP10-rfp-lys (red line), TOP10 bearing pHC-RFP induced with HSL 100 nM (green line) or uninduced (black line) are the negative controls. Induction was carried out in the exponential phase at OD600~0.55. Error bars represent the 95% confidence interval of the estimated mean.

Figure 5 A and B show respectively the OD600 during the experiment and the fluorescence of the supernatant, which is proportional to the RFP molecules released in the growth medium. It is evident that only TOP10-rfp-lys + HSL 100 nM could lyse and that cell lysis was accompanied by RFP release in the culture supernatant. For this culture, after 125 minutes from induction time, 96 ± 0.04% of the RFP molecules had been released in the growth medium (see Table 4). Surprisingly, the three negative control cultures released about 25% of the molecules.

**Table 4:** RFP release efficiency of TOP10-rfp-lys cultures grown at 37°C in 15 ml tubes and induced with HSL 100 nM

Strain	Secreted RFP [%]
TOP10-rfp-lys	24.84 ± 0.6
TOP10-rfp-lys + HSL 100 nM	95.95 ± 0.04
TOP10 with pHC-RFP	25.38 ± 1.8
TOP10 with pHC-RFP + HSL 100 nM	24.68 ± 0.8

Mean values estimated on three tubes in the same experiment are reported with standard errors.

### Analysis of mutants

**Figure 6:**
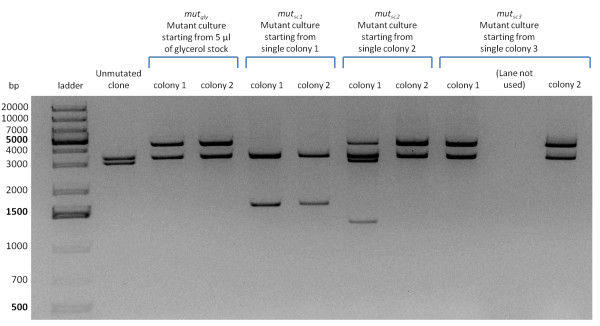
**Restriction analysis of pLC-T4LysHSL mutants**. Plasmid DNA was digested with EcoRI and PstI. In all the screened clones two bands (vector backbone and insert) are present except in colony 1 of mut_sc2_, in which four bands can be observed. The expected vector backbone (~3.2 kbp) is present in all the clones, demonstrating that deletions or insertions occurred only in the insert, while all the mutated insert bands are clearly different from the unmutated culture (~2.8 kbp). As sequencing showed, colony 1 of mut_sc2 _had plasmids with two different mutated inserts in the same clone, one of which containing an EcoRI restriction site that, when digested, produces 2 bands.

**Figure 7:**
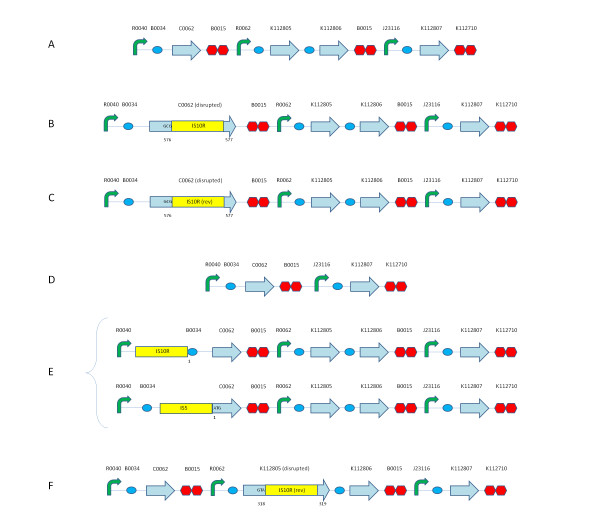
**Mutations occurred in the insert of pLC-T4LysHSL in mutant clones**. Unmutated BBa_K173015 (A); *luxR *disruption mediated by IS10R (B); *luxR *disruption mediated by IS10R (insertion in reverse direction relative to (B)) (C); deletion of the DNA fragment flanked by BBa_B0015, containing the *lux *promoter and the holin and lysozyme genes (D); two different mutated plasmids in the same clone: insertion of IS10R and IS5 (containing an EcoRI restriction site) upstream of the first nucleotide of BBa_B0034 RBS and *luxR*, respectively (E); *t *gene disruption mediated by IS10R (insertion in reverse direction relative to (B)) (F). The number under the parts denotes the nucleotide of the basic part flanking the insertion sequence that has disrupted the part. The disrupted genes are *luxR *(BBa_C0062), encoding the transcriptional activator of *lux *promoter (BBa_R0062), and *t *(BBa_K112805), encoding the holin. Part codes are given according to the Registry of Standard Biological Parts.

After performing lysis assays, the lysed cultures grew again (see Lysis assays section). When the re-grown cultures of TOP10 bearing pLC-T4LysHSL were diluted 1:1000 in fresh selective LB medium and let grow to an OD600 = 0.35 (exponential phase), they did not lyse upon induction with HSL 10 nM, suggesting that the cells have completely lost the inducible lysis phenotype (data not shown). The restriction analysis of the mutant plasmids after DNA digestion with EcoRI-PstI is reported in Figure 6. Plasmids were purified from two single colonies isolated from four mutant cultures. One of these cultures was obtained starting from 5 μl of glycerol stock (here called mut_gly _culture), while the other three cultures were obtained starting from single colonies streaked from the glycerol stock (here called mut_sc1_, mut_sc2 _and mut_sc3 _cultures respectively). In all the mutant clones the pSB4C5 vector band (~3.2 kbp) was present, but the insert was highly different from the unmutated culture (~2.8 kbp), suggesting that mutations occurred just in the HSL-inducible lysis device. The sequencing of the clones disclosed all the mutations, as shown in Figure 7. Two clones (colony 2 of mut_gly _and colony 2 of mut_sc3_) showed the insertion sequence IS10R after the nucleotide 576 of *luxR *(BBa_C0062) gene (Figure 7B); two clones (colony 2 of mut_sc2 _and colony 1 of mut_sc3_) showed the insertion of IS10R in the same place as before, but in the opposite direction (Figure 7C); two clones (colony 1 and 2 of mut_sc1_) showed the deletion of the DNA comprising the *lux *promoter and the holin-lysozyme operon (Figure 7D); one clone (colony 1 of mut_gly_) showed the insertion of IS10R after the nucleotide 318 of the holin (BBa_K112805) gene (Figure 7E); one clone (colony 1 of mut_sc2_) had two different mutated plasmids in the same colony: one of them showed the insertion of IS10R before the nucleotide 1 of the *luxR *RBS (BBa_B0034), while the other one showed the insertion of IS5 before the start codon of *luxR *(Figure 7F). All the described mutations cause the impairment of the lysis phenotype. The molecular weight of the restriction fragments shown in Figure 6 are all consistent with the found mutations.

### Evolutionary stability of bacteria bearing pLC-T4LysHSL

In order to study the reliability of the lysis device when cell disruption is not triggered, bacteria bearing pLC-T4LysHSL were propagated for 100 generations without HSL and their inducible lysis phenotype was tested over time in terms of lysis entity. Supplementary Figure 3 (Additional File 1) shows the results of this study in TOP10, DH5alpha and MG1655 grown in selective LB. Only MG1655 could maintain the phenotype of interest for 100 generations, thus showing an excellent stability of the uninduced lysis genes. Among these strains, TOP10 was the first to lose the lysis capability, which started to decrease after 30 generations and it was completely lost after 60. DH5alpha showed a high lysis entity variability because two of the three replicates completely lost the lysis capability after 60 generations, while the other one never lost it even in 100 generations, thus giving very wide standard errors of the measured mean.

## Conclusions

In this work, the quantitative characterization of a BioBrick™ lysis device of the Registry of Standard Biological Parts is reported. Its activity has been measured in *E. coli *using a well characterized HSL-inducible promoter and the transfer function, lysis dynamics, protein release capability, modularity and genotypic and phenotypic stability of the device have been evaluated.

Low copy number has been found to be the optimal working condition, as lysis could be triggered in all the growth phases of the bacterial culture and the cells grew with a relatively low metabolic burden, according to their doubling time. Lysis entity in late stationary phase was lower than in the other growth phases. These results are consistent with the published findings for which *E. coli *membrane disruption mediated by T4 phage lysis gene *t *or *e *was more efficient when the genes were expressed in the exponential growth phase than in the stationary phase, where less or almost no disruption occurred [8,11,13]. 96% of the total amount of intracellular proteins was successfully released into the growth medium upon induction of the lysis device in low copy plasmid. These features demonstrate that in this condition the expression of the lysis genes is tightly controlled and makes the device suitable for recombinant protein release upon gentle disruption of cell membranes. However, in all the experiments mutant cells that were unresponsive to induction arose after the bacterial lysis. This intrinsic instability makes the device unsuitable for the programmed cell death of a bacterial population. Mutant analysis showed that two main classes of DNA modifications occurred to eliminate the HSL-inducible lysis phenotype. The most frequent one consisted in the insertion of IS10R or IS5 within the device sequence to impair the expression of the lysis genes by disrupting their regulatory parts upstream or the lysis genes themselves. The other class exhibited the deletion of genes included between two identical DNA sequences. In particular, the DNA fragment including the *lux *promoter and the holin and lysozyme genes was deleted from the plasmid most probably by a replication slippage mechanism between two identical transcriptional terminators (BBa_B0015) of 129 bp flanking the fragment. These mutations were consistent with published DNA mutations commonly occurring in *E. coli *[22], in fact gene disruptions mediated by insertion sequences, which occurred in the majority of the mutant cultures analyzed in this work, have been found to be responsible of 95% of the mutation events in TOP10 strain [23]. On the other hand, replication slippage events have already been found to occur between two BBa_B0015 terminators in a previously studied BioBrick™ device [24]. The HSL-inducible lysis device stability was also studied during continual bacterial growth for 100 generations without induction. The inducible lysis phenotype started decreasing after only 30 generations and was completely lost after 60, but DH5alpha and MG1655 strains gave a better performance. In particular, DH5alpha showed a significantly lower lysis capability after 60 generations, while MG1655 stably maintained it during all the 100 generations. The described lysis device has been shown to be compatible with other modular input devices. When assembled with a heat-inducible BioBrick™ device upstream and triggered with a temperature shift to 42°C, the lysis device worked as expected, thus demonstrating the possibility of triggering cell lysis with any transcription-based input device. This feature enables the intriguing possibility to control cell disruption in response to a user-defined exogenous signal.

All the results have been confirmed in different *E. coli *strains and different growth media, thus providing parameters that can be used in models to aid future biological systems design and to facilitate the re-usability of this lysis device.

The lysis device in high copy number plasmid gave worse performance, in fact lysis entity was lower than in low copy plasmid, the metabolic burden was much higher and the device was strongly unstable, as cell lysis induction usually failed to occur.

## Competing interests

The authors declare that they have no competing interests.

## Authors' contributions

LP, SZ, ML and PM designed the experiments. LP, SZ and ML performed all the plasmid constructions and validation experiments. LP, SZ and PM analyzed the data. LP, MGCDA and PM wrote the manuscript. All authors read and approved the final manuscript.

## Endnotes

^1 ^BioBrick™ is a trademark of The BioBricks Foundation. (http://www.biobricks.org)
